# Supplementary material for: Translational Modeling Identifies Synergy between Nanoparticle-Delivered miRNA-22 and Standard-of-Care Drugs in Triple-Negative Breast Cancer
Source: Pharm Res. 2022 Mar 16;39(3):511–28. doi: 10.1007/s11095-022-03176-3 (PMC8986735; doi:10.1007/s11095-022-03176-3)

Supplementary Information

Translational Modeling Identifies Synergism between Nanoparticle-Delivered miRNA-22 and Standard-of-Care Drugs in Triple-Negative Breast Cancer

Prashant Dogra, Javier Ruiz Ramírez, Joseph D. Butner, Maria J. Peláez, Caroline Chung, Anupama Hooda-Nehra, Renata Pasqualini, Wadih Arap, Vittorio Cristini, George A. Calin, Bulent Ozpolat, Zhihui Wang*

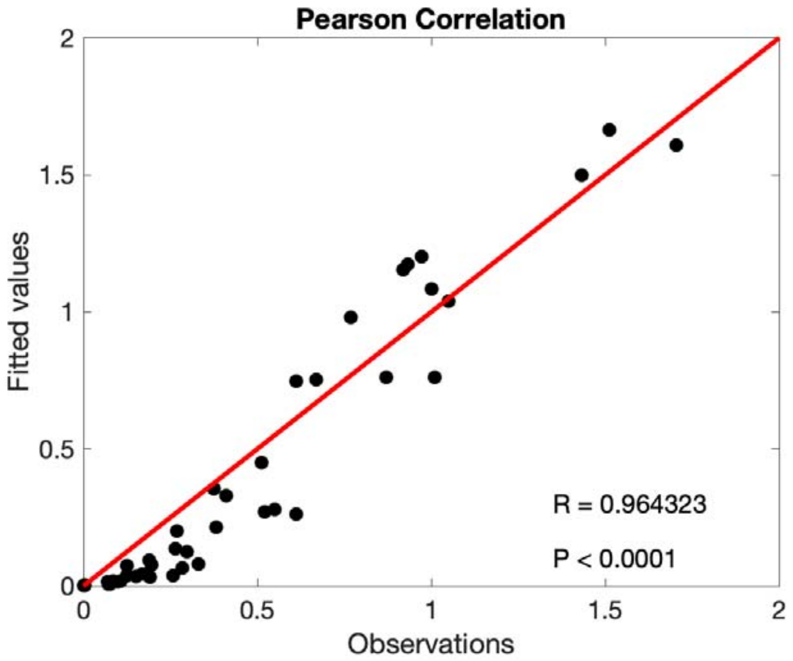

Figure S1. Pearson correlation of model fits to *in vivo* data shown in Figure 2.

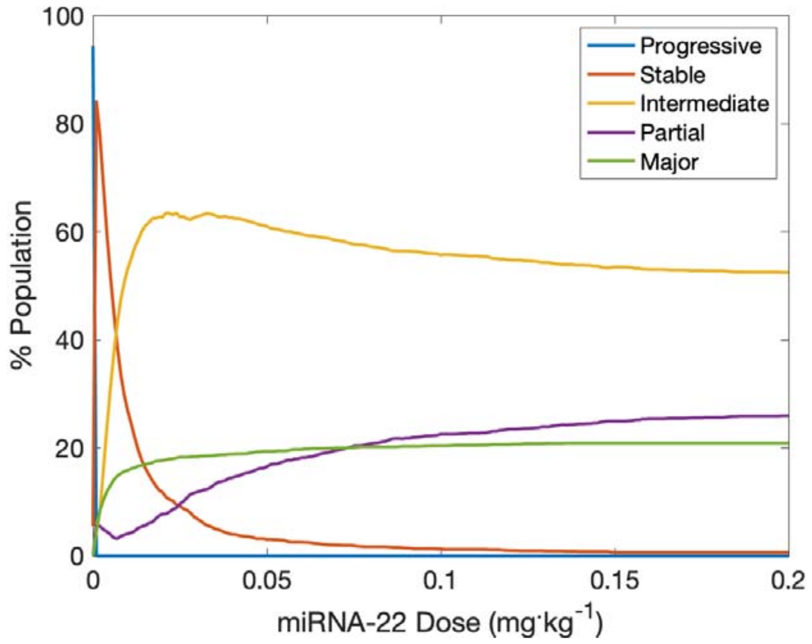

Figure S2. Population-scale dose-response relationship of miRNA-22 therapy.

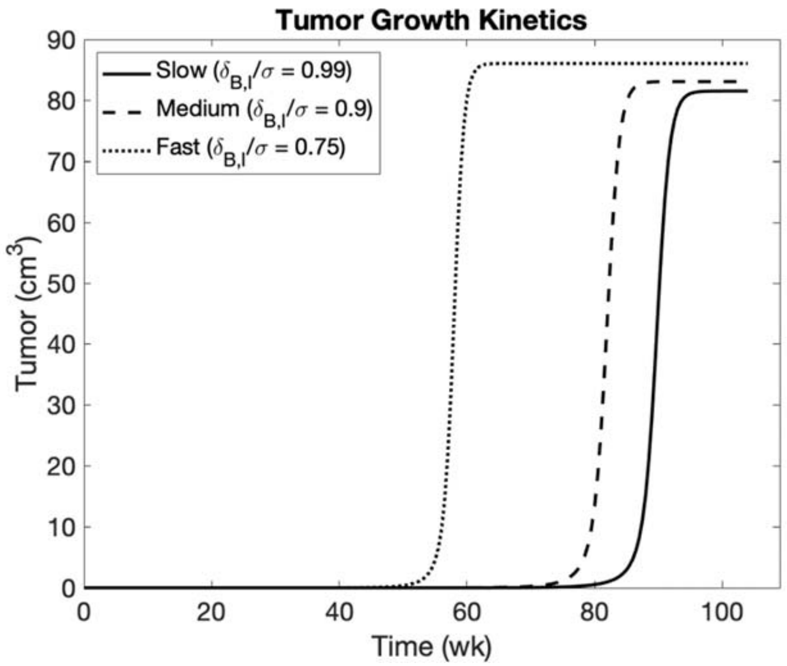

Figure S3. Tumor growth kinetics of slow-, medium-, and fast-growing tumors under the control conditions.

CompuSyn Report

**Experiment Name:**	AAPS paper
**Date:**	7/14/21
**File Name:**	C:\Users\prash\Desktop\report.cse
**Description**	

Drug: mirna22 (mirna) [mg/kg]
Drug: dox (dox) [mg/kg]
Drug: ate (ate) [mg/kg]
Drug Combo: mirna+dox (mirdox) (mirna+dox)
Drug Combo: mirna+ate (mirate) (mirna+ate)
Drug Combo: mirnadoxate (mda) (mirna+dox+ate)

Data for Drug: mirna [mg/kg]

Dose	Effect
0.01	0.2017
0.0311	0.3087
0.0522	0.3466
0.0733	0.3662
0.0944	0.3782
0.1156	0.3864
0.1367	0.3922
0.1578	0.3966
0.1789	0.4001
0.2	0.4029

10 data points entered.
X-int: -0.2695
Y-int: 0.08429 +/- 0.03407
m: 0.31277 +/- 0.02925
Dm: 0.53764
r: 0.96676

Data for Drug: dox [mg/kg]

Dose	Effect
0.01	0.0012
1.12	0.0589
2.23	0.087
3.34	0.1068
4.45	0.1225
5.56	0.1352
6.67	0.146
7.78	0.1553
8.89	0.1635

Dose	Effect
10.0	0.1708

10 data points entered.

X-int: 1.81569

Y-int: -1.3608 +/- 0.02420

m: 0.74946 +/- 0.02588

Dm: 65.4173

r: 0.99526

Data for Drug: ate [mg/kg]

Dose	Effect
0.01	0.08402
1.12	0.08454
2.23	0.08454
3.34	0.08455
4.45	0.08455
5.56	0.08455
6.67	0.08455
7.78	0.08455
8.89	0.08455
10.0	0.08455

10 data points entered.

X-int: 1033.99

Y-int: -1.0352 +/- 1.13E-4

m: 0.00100 +/- 1.20E-4

Dm: Infinit

r: 0.94670

Data for Non-Constant Combo: mirdox (mirna+dox)

Dose mirna	Dose dox	Effect
0.01	0.01	0.2064
0.0311	1.12	0.5982
0.0522	2.23	0.7995
0.0733	3.34	0.8892
0.0944	4.45	0.9291
0.1156	5.56	0.9493
0.1367	6.67	0.9609
0.1578	7.78	0.9683
0.1789	8.89	0.9733
0.2	10.0	0.9769

10 data points entered.

Data for Non-Constant Combo: mirate (mirna+ate)

Dose mirna	Dose ate	Effect
0.01	0.01	0.32318

Dose mirna	Dose ate	Effect
0.0311	1.12	0.44847
0.0522	2.23	0.49304
0.0733	3.34	0.51610
0.0944	4.45	0.53018
0.1156	5.56	0.53967
0.1367	6.67	0.54648
0.1578	7.78	0.55162
0.1789	8.89	0.55563
0.2	10.0	0.55885

10 data points entered.

Data for Non-Constant Combo: mda (mirna+dox+ate)

Dose mirna	Dose dox	Dose ate	Effect
0.01	0.01	0.01	0.32890
0.0311	1.12	1.12	0.83052
0.0522	2.23	2.23	0.95271
0.0733	3.34	3.34	0.97687
0.0944	4.45	4.45	0.98529
0.1156	5.56	5.56	0.98933
0.1367	6.67	6.67	0.99164
0.1578	7.78	7.78	0.99312
0.1789	8.89	8.89	0.99414
0.2	10.0	10.0	0.99488

10 data points entered.

Median-Effect Plot for Drugs

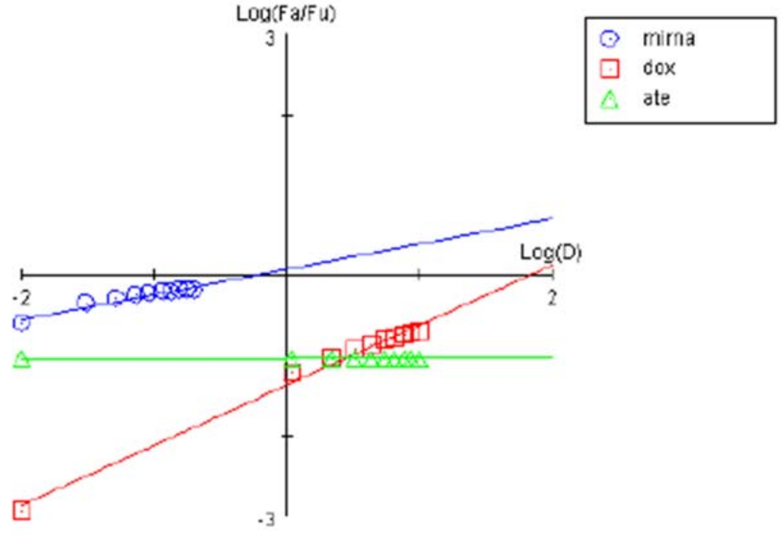

Median-Effect Plot for Drug Combos

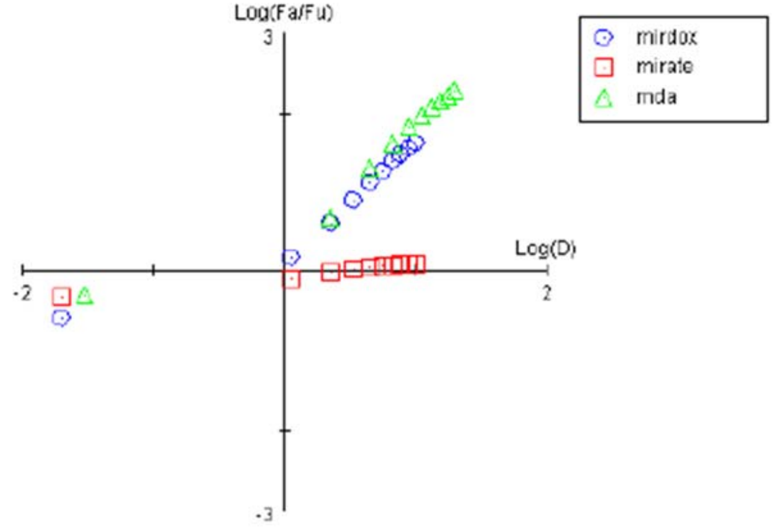

CI Data for Non-Constant Combo: mirdox (mirna+dox)

Dose mirna	Dose dox	Effect	CI
0.01	0.01	0.2064	1.37989
0.0311	1.12	0.5982	0.02627
0.0522	2.23	0.7995	0.00655
0.0733	3.34	0.8892	0.00335
0.0944	4.45	0.9291	0.00224
0.1156	5.56	0.9493	0.00172
0.1367	6.67	0.9609	0.00143
0.1578	7.78	0.9683	0.00125
0.1789	8.89	0.9733	0.00112
0.2	10.0	0.9769	0.00104

CI Data for Non-Constant Combo: mirate (mirna+ate)

Dose mirna	Dose ate	Effect	CI
0.01	0.01	0.32318	0.19766
0.0311	1.12	0.44847	0.11207
0.0522	2.23	0.49304	0.10613
0.0733	3.34	0.51610	0.11095
0.0944	4.45	0.53018	0.11930
0.1156	5.56	0.53967	0.12933
0.1367	6.67	0.54648	0.14007
0.1578	7.78	0.55162	0.15131
0.1789	8.89	0.55563	0.16287
0.2	10.0	0.55885	0.17465

CI Data for Non-Constant Combo: mda (mirna+dox+ate)

Dose mirna	Dose dox	Dose ate	Effect	CI
0.01	0.01	0.01	0.32890	0.18226
0.0311	1.12	1.12	0.83052	0.00241
0.0522	2.23	2.23	0.95271	6.27E-4
0.0733	3.34	3.34	0.97687	3.47E-4
0.0944	4.45	4.45	0.98529	2.49E-4
0.1156	5.56	5.56	0.98933	2.02E-4
0.1367	6.67	6.67	0.99164	1.74E-4
0.1578	7.78	7.78	0.99312	1.56E-4
0.1789	8.89	8.89	0.99414	1.44E-4
0.2	10.0	10.0	0.99488	1.35E-4

Combination Index Plot

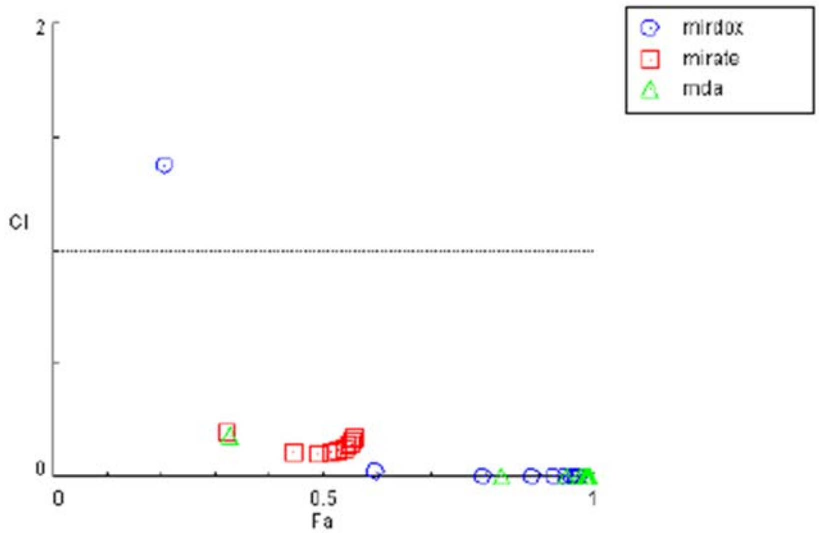

Supplement: Supplementary file 1 — (PDF 599 kb) [file 11095_2022_3176_MOESM1_ESM.pdf]
